# Impact of common interest group participation and aquaculture development programs on fish productivity and net returns: evidence from Nile tilapia farming

**DOI:** 10.1007/s10499-024-01707-w

**Published:** 2024-11-14

**Authors:** Castro N. Gichuki, S. Wagura Ndiritu, Achom Barbara Emodoi

**Affiliations:** Strathmore Agri-Food Innovation Center, Strathmore University Business School, Nairobi, Kenya

**Keywords:** Development programs, Genus tilapia, Common interest groups, Small holder farmers

## Abstract

Development programs have intensified support for the aquaculture sector to improve production and productivity, as well as food security and diet quality for smallholder farmers. This paper examines the impact of farmers’ participation in Common Interest Groups (CIGs) and the effects of aquaculture development support programs on the net returns and productivity of tilapia fish in Kenya. The study used household-level data of 506 members of the CIG who were randomly selected for the treatment group and benefited from Aquaculture Business Development Program (ABDP) interventions. The analysis employed an endogenous switching regression model to account for selection bias. The results reveal that membership in CIGs and receiving fish production interventions have resulted in a 32.3% increase in tilapia fish sales returns and a 6.6% increase in tilapia fish yields. Specifically, ABDP interventions are aimed at providing fish producers with ponds and cages had a significant and beneficial effect on fish yields and income returns. This finding suggests that policies with targeted interventions that support aquaculture infrastructure can play a significant role in increasing the incomes of smallholder farmers and reducing rural poverty in Kenya.

## Introduction

Around 800 million individuals residing in developing nations experience food insecurity, with 25% of them originating from Sub-Saharan Africa (SSA) (Aryal et al. 2022). Between 2012 and 2014, over 214 million people, accounting for over one-third (23.8%) of the population, experienced undernourishment. This means that they were consuming less than the accepted daily caloric intake threshold of 2350 kcal/day (Sullivan and Hickel 2023). Furthermore, more than 300 million people (49%) live in poverty (earning less than one dollar a day) (Moatsos and Lazopoulos 2021). SSA will not achieve the second Sustainable Development Goals (SDGs) of reducing global hunger by 50% by 2030 if agriculture, agricultural food production, targets, and support interventions are not well established (Atukunda et al. 2021; de Pryck 2013). Notwithstanding the challenges, the fishing and aquaculture sector can play a significant role in driving economic activity across SSA countries.

The sector provide employment, livelihood, community development, and food security, as well as alleviating poverty and supplying a substantial share of animal protein (Mathewos et al. 2019). At present, approximately 12.3 million individuals are employed in the SSA fisheries sector; of these, half are fishermen, 42.4% are processors, and 7.5% are fish farm laborers (Muringai et al. 2021). Nevertheless, Africa contributed only 7% to the total fisheries and aquaculture production as compared to Asian countries who are the leading producers at 70%, the Americas 12%, Europe 10%, and 15% from Oceania (Food Agriculture Organization 2022).

In SSA, fish is mainly derived from two sources: capture and culture. The capture fishery is a crucial activity that plays a significant role in promoting sustainability and broad-based development in SSA (Majumdar et al. 2023). Capture fisheries, while still the primary source of fish supply, seems to have stagnated or be on the declined as a result of overfishing and environmental degradation and it is currently thought that aquaculture has the best potential to fill the increasing demand for fish from the growing population in the region(Muringai et al. 2021). Fish catches from the lake and other freshwater lakes have decreased (Outa et al. 2020). In SSA, lakes that have been a major source of fish capture are currently facing a number of issues, including water hyacinth invasion, biodiversity loss, eutrophication, and pollution, among others (Maina et al. 2014). However, aquaculture farming is acknowledged to have increased fish supplies globally during the previous three decades and has potential to grow, as most catch fisheries have been fully or overexploited (Naylor et al. 2021).

Aquaculture farming is the fastest-growing food-producing industry, surpassing both farm animal meat production and landings from capture fisheries (Kumar et al. 2013). The aquaculture sector in Africa experienced a significant growth in production, increasing by twenty times from 110,200 to 2,196,000 tons between 1995 and 2018 (Adeleke et al. 2020). This is a compound annual growth rate of approximately 16%. Additionally, the contribution of aquaculture to total fish production by weight rose from 6.2% in 2000 to 18.5% in 2017 (Munguti et al. 2023). In SSA, the majority (99%) of the aquaculture production comes from inland freshwater systems and is primarily focused on cultivating native and abundant species such as tilapia and African catfish (Food Agriculture Organization 2022). Mariculture, on the other hand, only accounts for a small portion (1%) of the total production, despite being a growing and promising subsector.

Tilapia occupies a distinctive position among the principal aquaculture fishes in that it is produced in substantial quantities as a subsistence crop by some of the world’s poorest farmers while also serving as a key product in international commerce through the operation of large vertically integrated farming operations. Due to their exceptional hardiness and mouth-brooding mode of reproduction, tilapias provide an opportunity for producers with limited resources to cultivate the fish in SSA (Majumdar et al. 2023). Furthermore, tilapia exhibits favorable characteristics for aquaculture production including a brief life cycle, rapid growth rate, and minimal input requirements. Tilapia demonstrates considerable potential as an aquaculture species in appropriate seasonal habitat (Saha et al. 2022).

The exponential population growth puts strain on cultured fish species, particularly tilapia. As a result, increasing cultivable land for fish necessitates making efficient use of available resources. Therefore, increased demand needs to be met by similarly rapid supply growth; fish shortages are expected to result in decreasing fish consumption, particularly among the poor, and pose a danger to food security (Mitra et al. 2023). Enhancing the efficiency of tilapia smallholder farmers through the use of existing technologies can enhance tilapia production and encourage them to profit (Khan et al. 2021).

Globally, small-scale fish farmers play a vital role in supplying marine protein to consumers in developing countries. In SSA, small-scale fish farmer (SSFF) is crucial for maintaining food security, nutrition, and livelihoods in urban and rural areas (Short et al. 2021). These farmers are crucial in supplying protein, generating income, and providing employment for numerous households in developing nations, making them vital for poverty alleviation, nutritional security, and job creation in supporting millions of people across Africa (Giller, et al. 2021; N'Souvi, et al. 2023). Their impact is particularly significant in communities where economic opportunities beyond farming are scarce, underscoring the critical role of small-scale fisheries (Sowman and Cardoso 2010). Moreover, as a result of its low market price, fish has emerged as a vital food source for impoverished (Henry-Silva et al. 2022). However, despite their critical contribution to both local and national economies, the aquaculture production sector often faces challenges such as inadequate planning, regulation, underfunding, and marginalization compared to other sectors of the food economy (Kaminski et al. 2020).

Despite the growing demand for fish products, many small-scale fish farmers struggle with production inefficiencies and face the challenge of declining market prices. In addition to the obstacle posed by declining output prices, producers’ profits have been impacted in recent years by rising feed costs and deteriorating feed quality (Musa et al. 2022). As a result of the combined impact of the high cost of inputs and the low market value of tilapia, certain farms are operating at a loss rather than generating a profit (Atukunda et al. 2021; Stutzman et al. 2017). The significant growth potential of the aquaculture sector has captured the interest of governments, private businesses, philanthropic organizations, and international conservation groups (Kaminski et al. 2020). This has led to increased investments in new technologies and scientific management practices, which promise higher returns at lower costs. Advancements in these technologies and production systems are closely linked, with research enhancing traditional knowledge to improve fish breeding and feeding techniques (Jayne et al. 2021).

In SSA, governments and development agencies such as World Bank and Food Agriculture Organization (FAO) have doubled the efforts to promote aquaculture farming, develop fisheries resources while promoting investments in the blue economy sector. The efforts are aimed at improving productivity and incomes of SSFF as well as the food security and diet quality among the poor households in rural and urban towns (Odende et al. 2022). In Kenya, the government and International Fund for Agricultural Development (IFAD) partnered to support SSSF through Aquaculture business development program (ABDP) (Munguti et al. 2023). ABDP aims to support the growth of the aquaculture industry by implementing measures that focused on the establishment of fish farming ponds, restoration of current production ponds, provision of training and skill development for SSFF, and promotion of technical and business expertise.

Utilizing the ADBP project, this paper examined the impact of farmers' participation in Common Interest Groups (CIGs) and the effects of aquaculture development support programs on the net returns and productivity of tilapia fish in Kenya. The study uses a unique survey dataset that includes interviews with both smallholder fish farmers involved in CIGs and those who are not. The survey collected information on varying issues such as fish production and marketing, knowledge and use of sustainable aquaculture technologies, sources of information on aquaculture practices, and access to government support. The survey data was gathered under the Green Venture Africa project, which examined challenges such as fragmented markets, low purchasing power, insufficient government support, risk aversion, and limited diversity in capital sources that hinder the scaling of aquaculture agribusiness in Kenya.

The study makes two contributions to literature. First, it contributes by exploring the causal impact of government supported aquaculture programs inventions such as aquaculture technologies (commercial fish feeds, breeding and genetic techniques, value addition, and post-harvest loss reduction techniques) influence on farmers’ incomes and yields. Numerous researchers have explored how aquaculture development affects socio-economic well-being, particularly concerning household food security. For example, Munene and Wanjiku (2020) analyzed the influence of aquaculture development on food security, while Garlock et al. (2022) concentrated on the primary factors that enable fish farming to create livelihoods and alleviate poverty. A study by Obwanga et al. (2020) emphasizes the significance of access to various capital assets human, social, natural, physical, and financial and the importance of diverse transformative processes, including markets, institutions, facilities, infrastructure, and services.

The second contribution to the expanding literature emphasizes the significance of common interest groups as vital rural farming institutions that facilitate the dissemination of agricultural information and serve as channels for implementing government programs. While the adoption of aquaculture technologies among smallholder farmers is well-advanced in Asia, it remains underdeveloped in Sub-Saharan Africa (SSA) (Mafwila Kinkela et al. 2019; Odende et al. 2022; Tezzo et al. 2021). Studies by Obwanga et al. (2020) in Kenya and Ragasa et al. (2018) in Ghana indicate a low uptake of aquaculture technologies, attributed to gaps in best management practices and technical skills, which this study addresses. Additionally, in rural Bangladesh, non-governmental organizations have involved women in aquaculture programs to enhance incomes and food nutrition. Pucher et al. (2013) and Tri et al. (2021) explored similar programs in Vietnam, aiming to promote polyculture fish production and increase commercial fish production. However, farmer membership in common interest groups may significantly influence participation in development programs that support aquaculture technology adoption, thereby boosting returns and incomes. The remainder of the paper is organized as follows: “Methods” provides an overview of the methods and sampling used in the study. “Empirical method” covers the theoretical and empirical specifications as well as the estimation techniques. “Results and discussion” includes a description of the data utilized and the empirical results obtained. The conclusions are discussed in the final section, “Conclusions and policy implications.”

## Methods

The data used in this paper was gathered from regions where the Aquaculture Business Development Programme (ABDP) was implemented. The ABDP was a collaborative effort between the Government of Kenya and the International Fund for Agricultural Development (IFAD). The initiative was aimed at enhancing the capabilities of smallholder farmers, primarily those managing aquaculture ponds, to boost production and productivity. ABDP offered interventions such as the establishment of aquaculture production facilities, which included ponds. Additionally, they provided training and capacity building in aquaculture cage management, technical and business skills, and training in feeding and fingerling breeding. The major recipients of ABDP program interventions are farmers who are members of CIGs.

The data utilized in this study was gathered from a farm household survey conducted in Kenya between September and October 2023. Trained research assistants collected data from fish farming households. A multistage sampling procedure was employed to select the households for the study. In the first stage, four counties—Homabay, Busia, Siaya, and Kisumu—were purposively chosen based on their geographic accessibility and their representation of the 15 counties where the ABDP was implemented. In the second stage, random sampling was used to select both CIG households that benefited from ABDP (treatment group) and Non-CIGs (comparison group) households from each county. In the third stage, a total of 724 small aquaculture farming households, consisting of 506 treatment and 218 comparison households, were randomly sampled in proportion to the fish farmer population in each county. Specifically, 182 households were selected from Siaya County, 182 from Busia County, 180 from Kisumu County, and 180 from Homabay County. Structured questionnaires were used to interview the sampled farming households. The information collected primarily covered fish farm and household characteristics, fish production, marketing, and the adoption of fish production technologies.

## Empirical method

### Theoretical model specification

This study followed the approach of Abdulai and Huffman (2014) and Ma and Abdulai (2016) theoretical approach. The study’s conceptual framework is based on random selection of aquaculture farmers classified in the treatment group that received project interventions, such as aquaculture training, agribusiness training, and support for pond and fingerling supplies and control group. In the study we assume that fish farmers are mostly risk neutral and take into account the potential net returns and incomes ${D}_{m}^{*}$ from the venture derived from fish production under CIG membership while ${D}_{n}^{*}$ represents the expected income returns from fish derived from Non-CIG membership (comparison group). If we define the difference between the expected incomes from being a CIG member and Non-CIG member as ${D}_{i}^{*}$, where ${D}_{i}^{*}={D}_{m}^{*}-{D}_{n}^{*}$, therefore a farmer would choose to belong to CIG if ${D}_{i}^{*}>0.$ In that case, ${D}_{i}^{*}$ that is not observed is expressed as a function of observable components denoted in the latent variable model:

1 $${D}_{i}^{*}={Z}_{i}\beta +{\mu }_{i},{D}_{i}=1 \text{if }{D}_{i}^{*}>0 \text{otherwise}.$$

where ${D}_{i}$ is a binary indicator variable that equals 1 for the household *i*, in case of CIG membership and zero otherwise. ${Z}_{i}$ is a vector of covariate for fish farmers household and aquaculture farm characteristics represented by age, education level household size, and pond size, while β is the vector of coefficients, while ${\mu }_{i}$ is an error term assumed to be normally distributed with a 0 mean. Therefore, the probability of being a CIG member is expressed as

2 $$\text{Pr}\left({D}_{i}=1\right)=\text{Pr}\left({D}_{i}^{*}>0\right)=\text{Pr}\left({\mu }_{i}>-{Z}_{i}\beta \right)=1-F\left(-{Z}_{i}\beta \right)=1-F\left({Z0}_{i}\beta \right),$$

where *F* is the cumulative distribution function (CDF) of the error term (${\mu }_{i}).$ In order to establish a connection between CIG member and the prospective outcome of benefiting from ABDP, we make the assumption that rational farmers aim to maximize the net returns (*p*) from fish production. Consequently, this can be formulated as.

3 $${\pi }_{max}=PQ\left(R,Z\right)-OR$$

*P* represents the price of fish, and *Q* represents the total quantity of fish produced. *O* is a vector containing input prices, whereas *R* represents a vector including input variables such as fingerlings and feeds. *Z* is a vector consisting of explanatory variables previously described. A well-behaved production function with positive first derivative: can be expressed as $\frac{{\partial }^{2 }Q}{{\partial }^{2}R}>0$. The marginal product of the input *R* is positive, meaning that increasing the input leads to an increase in output. Therefore, the input *R* contributes positively to output *Q.* Negative second derivative is expressed as $\frac{{\partial }^{2 }Q}{{\partial }^{2}R}<0$*.* The production function exhibits diminishing marginal returns, meaning that as more of input *R* is used, the additional output produced from each additional unit of* R* decreases. This reflects a concave relationship between input *R* and output *Q*.

The relationship between fish returns can be determined by considering input and output prices, the decision to join a CIG, and the characteristics of the household and aquaculture farm.

4 $$\pi =\pi (P,O,D,Z)$$

The maximizing problem of the net returns function (Eq. (3)) results in fish output supply function, as determined by the first-order requirement.

5 $$Q=Q(P,O,D,Z)$$

Equations (4) and (5) indicate the net returns from fish production (*p*) and the quantity of fish produced (*Q*) that are influenced by factors such as input and output pricing, the decision to join CIG and benefit from ABDP interventions and the characteristics of the household and aquaculture farm.

### ESR model specification

The ESR model consists of two stages. The first stage is a selection equation based on a dichotomous criterion function for the choice of decision to join CIG membership, as shown by Eq. (1). In the second stage, two regime equations for CIG membership (treatment group) and Non-CIG membership (comparison group) are represented by the following two regression equations can fall in the following two regression equations.

5a $$\text{Regime }1: {Y}_{1i}={\beta }_{1}{X}_{i}+{\varepsilon }_{1i} if {I}_{i}=1$$

5b $${\text{Regime }2: Y}_{2i}={\beta }_{2}{X}_{i}+{\varepsilon }_{2i} if {I}_{i}=0$$

where ${Y}_{1i}$ and ${Y}_{2i}$ are outcomes such as fish yields, net returns income from fish for CIG membership (treatment group) and Non-CIG members (comparison group), respectively. ${\beta }_{1}{\beta }_{2}$ are parameters that show the direction and strength of the relation between the outcome variable and the independent variables, while ${\varepsilon }_{1i}$ and ${\varepsilon }_{2i}$ are error terms. Multiple methodologies can be employed to estimate the endogenous switching model. The estimate process can utilize either two-step least squares or maximum likelihood estimation. This involves estimating one equation at a time (Lokshin and Sajaia 2004). However, these methods are considered inefficient and lead to heteroskedastic residuals, requiring complex adjustments to ensure consistent standard errors (Abdulai and Huffman 2014). To address this limitation, one might employ the Full Information Maximum Likelihood (FIML) method to estimate the model.

Aquaculture farmers’ decision to participate into CIG is based on the assumption that the net benefits from ABDP will result fish income and yields than that of not joining CIG. Several types of unobservable factors also determine the aquaculture farmers’ decision to join CIG resulting in a selection bias. Therefore, a selection bias arises if unobservable factors affect both error terms in the selection equation (*ui*) and the outcome equation (*ε*). This leads to a correlation between the error components of the selection and the continuous equation, denoted as corr (*ε*_*i*_,_*i*_) = ρ ≠ 0. The presence of endogenous switching is evidenced by the correlation between the error terms (Maddala 1986).

Let's assume that the error factors ${\varepsilon }_{1i}$, ${\varepsilon }_{2i}$, and *ui* in Eqs. 5a and 5b follow a trivariate normal distribution with a mean of zero and a covariance matrix. According to Lee (1982), this can be expressed by

6 $$Cov\left(\text{ui},\upvarepsilon 1\text{i},\upvarepsilon 2\text{i},\right)=\left[\begin{array}{ccc}{\sigma }_{u}^{2}& .& .\\ {\sigma }_{\varepsilon 1u}^{2}& {\sigma }_{\epsilon 1}^{2}& .\\ {\sigma }_{\epsilon 2u}^{2}& .& {\sigma }_{\epsilon 2}^{2}\end{array}\right]$$

The ${\sigma }_{u}^{2}$ variance of the error term in the selection equation is denoted by ${\sigma }_{\epsilon 1}^{2}$, whereas the variances of the error terms in the continuous equations are denoted by ${\sigma }_{\epsilon 2}^{2}$. The standard deviation of *u* and the covariance of *ui* and *ε*2*i* and *ε*1*i* and *ε*2*i*, respectively. As ${Y}_{1i}$ and ${Y}_{2i}$ are not observed at the same time, the covariance of the error terms cannot be determined (Maddala 1986). The correlation between the error terms of the outcome equation and the selection equation leads to a non-zero anticipated value of $\upvarepsilon 1\text{i}$ and $\upvarepsilon 2\text{i}$ given *ui*—error term of the selection equation (Abdulai and Huffman 2014).

7 $$\begin{array}{c}E\left({\varepsilon }_{1}|I=1\right)\text{and }E\left({\varepsilon }_{2}|i=0\right)\text{given by}\\ \begin{array}{c}E({\varepsilon }_{1}|I=1)= E({\varepsilon }_{1}|u>-Z\propto )={\sigma }_{\varepsilon 1u} \frac{\phi (\frac{Z\alpha }{\sigma })}{\Phi (\frac{Z\alpha }{\sigma })}\equiv {\sigma }_{\varepsilon 1u}{\lambda }_{1\text{ a})}\\ E\left({\varepsilon }_{2}|I=0\right)=E({\varepsilon }_{2}|u\le Z\propto ) ={\sigma }_{\varepsilon 2u}\frac{-\phi \left(\frac{Z\propto }{\sigma }\right)}{1-\Phi (\frac{Z\propto }{\sigma })}\equiv {\sigma }_{\varepsilon 2u}{\lambda }_{2}\end{array}\end{array}$$

$\phi$ and *Φ* represent the probability density and cumulative distribution function of the standard normal distribution, respectively. The inverse Mills ratio, denoted as λ1 and λ2, is the ratio of $\phi$ and Φ when assessed at Zα. These terms are commonly known as selectivity terms. If the calculated covariance σε21*u* and σε22*u* deviate significantly from 0, there exists a correlation between the decision to join CIG and the outcome variable (fish returns). This indicates the occurrence of endogenous switching and the existence of a sample selectivity bias (Maddala and Nelson 1975).

The correlation coefficients ρ1 and ρ2 represent the relationship between the error term *ui* in the selection equation and the error terms ε1 and ε2 in the outcome equations. In addition, treatment effects were estimated. The Average Treatment Effect on the Treated (ATT) and Average Treatment Effect on the Untreated (ATU) are calculated by comparing the expected values of the dependent variable for those who received the treatment and those who did not, in both the actual and counterfactual circumstances.

8 $$\begin{array}{c}E\left({Y}_{1i}|{I}_{i}=1,{X}_{1i}\right)={\beta }_{1}{X}_{1i}+{\sigma }_{\epsilon 1u\rho 1}\frac{\varphi (Z\propto )}{\Phi (Z\propto )}\\ E\left({Y}_{2i}|{I}_{i}=0,{X}_{2i}\right)={\beta }_{1}{X}_{2i}-{\sigma }_{\epsilon 2u\rho 1}\frac{\varphi (Z\propto )}{(1-\Phi (Z\propto ))}\\ \begin{array}{c}E\left({Y}_{2i}|{I}_{i}=1,{X}_{1i}\right)={\beta }_{2}{X}_{1i}+{\sigma }_{\epsilon 2u\rho 2}\frac{\varphi (Z\propto )}{\Phi (Z\propto )}\\ E\left({Y}_{1i}|{I}_{i}=0,{X}_{2i}\right)={\beta }_{1}{X}_{2i}-{\sigma }_{\epsilon 2u\rho 2}\frac{\varphi (Z\propto )}{(1-\Phi Z\propto ))}\end{array}\end{array}$$

The Average Treatment Effect (ATT) is the discrepancy between the anticipated value of the outcome variable derived from Eq. (6) and Eq. (8). The difference lies in the predicted value of the dependent variable for users who have utilized it compared to those who have not. The ATU (Average Treatment Effect on the Untreated) is the discrepancy between Eqs. 7 and 9, which serves as an estimation of the disparity between the anticipated value of the outcome variable for individuals who do not use the water and the hypothetical scenario where they do use the water.

#### Addressing potential endogeneity

For the ESR model to be properly identified, the ${Z}_{i}$ variables in Eq. (2) must include at least one selection instrument—this is a variable that significantly influences the selection process but does not directly impact the outcome variable (Ndiritu and Muricho 2021). As a result, one instrument is excluded from the estimation in Eq. (1). Furthermore, for valid identification, the two instrumental variables must not be correlated with each other (Rivers and Vuong 1988). Therefore, in the study, extension access and trust in farmers groups perceptions are selected as instrumental variable. Agricultural extension agents may introduce agribusiness training to farmers, therefore, making extension access potentially endogenous. The perception of trust in farmers groups can be endogenous to a farmer’s choice to join a CIG due to self-selection, reverse causality, unobserved factors, peer influence, and cultural norms.

A past empirical study by Ma and Abdulai (2016) hypothesized that the perception of the usefulness of extension and endogenous as strongly influence the given potential endogenous variables, choice of cooperative membership. Farmers' perceptions of trust processes in the groups influenced their decision to remain in or join these groups, reinforcing the idea that perception can be endogenous to the participation decision (Barham and Chitemi 2009). This method involves specifying the potentially endogenous variables (extension access and trust in farmers groups) as functions of all other explanatory variables in the CIGs membership choice equation, alongside a set of instruments used in the first-stage regression, such as:

9 $${G}_{i}={Z}_{i}\beta +{S}_{i}\omega +{\mathfrak{I}}_{i}$$

where ${G}_{i}$ represents a vector of observed potential endogenous variables, such as extension access and the production cycle, ${Z}_{i}$ represents the previously defined variables, and ${S}_{i}$ represents a vector of instruments. It is important that the instruments used strongly influence the potential endogenous variables but not the choice of CIG membership. Finally, in the CIGs membership choice specification, we incorporate both the observed factors, and the predicted residuals are presented in Eq. (10) in the following manner:

10 $${D}_{i}^{*}={Z}_{i}\delta +{G}_{i}\eta +{R}_{i}K+{\upsilon }_{i}$$

${R}_{i}$ is a vector of the residual terms predicted from Eq. (8) for the endogenous variables (Semykina and Wooldridge 2010). By incorporating these residuals into the second stage estimating equation, the endogenous variables become appropriately exogenous, as the residuals act as control functions. This method results in a robust regression based on Hausman test for detecting the endogeneity of the suspected variables (Wooldridge 2015).

## Results and discussions

### Sampled farming household characteristics

Table 1 presents information on the definition and summary statistics of the variables used for the empirical analysis. The data reveal that 69% of farmers in the sample were CIGs members that mainly included aquaculture training and investment pod facilities. The remaining 31% of farmers did not benefit from the ABDP programme. As shown in Table 1, the average age of a farmer is 51 years with an average of about 13.6 years of formal education. Majority of the households sampled (88%) were male headed, and the households mainly comprised of 6 household beneficiaries. On average the farmers owned two fishponds and cages averaged 478.3 m^3^, showing that the majority of households are small-scale fish producers. The data also reveal that on average, the fish farmers harvested 3680.3 kg of fish and earned returns of 73676.3 Ksh from fish sells annually.

**Table 1:** Definition and summary statistics of selected variables

Variable	Definition	Mean (sd)
Beneficiary of (ABDP)	1 If farmer benefited from ABDP, 0 otherwise	0.6979
Fish yields at harvest (kg)	Annual fish output at harvest (kg/yr)	3680.35 (118.1)
Returns from fish sells (Ksh)	Household income from fish sells (Ksh)	73676.37 (113.8)
Household size	Number of people residing in the household	6.0883(2.8020)
Age	Age of respondent (years)	51.31 (13.71)
Education	Highest years of schooling of the household(years)	13.621 (2.910)
Gender	1 if farmer is male, 0 other wise	0.8897
Dist to main fish market	Distance from the household to main fish farm in (Km)	1.7471
Credit	1 if farmer accede credit for fish production, 0 other wise	0.4690
Ponds	Number of fish production ponds owned	2.377 (3.352)
Cage size	Size of the cage facility (square meters)	478.3 (2359)
Membership to Cooperatives	1 if farmer belongs to Co-operative, 0 otherwise	0.601
Selective breeding	1 if farmer has adopted selective breeding technology, 0 otherwise	0.6823
Feed regime	1 if farmer has adopted feed regime technology, 0 otherwise	0.9476
Hormonal sex reversal	1 if farmer has adopted hormonal sex reversal technology, 0 otherwise	0.5732
Support from crop farming Projects	1 if farmer benefited from crop farming project support project, 0 otherwise	0.3903
Extension access	1 if farmer received extension on fish production and business training, 0 otherwise	0.736
Fingerlings stocked	Number of fingerlings stocked in cages	2894.4 (2929.3)
Production cycle	Production cycle of tilapia in months	8.184 (1.668)

Ksh is Kenyan currency (US$1 = 130 Ksh)

The variable mean differences and associated statistical t tests between CIG members and Non-CIG households are presented in Table 2. The results reveal significant mean differences in household fish returns between CIGs members and non- members. CIG members generated Ksh 81,514.7 (632.88 USD) returns from fish sales, whereas Non-CIG household generated Ksh 55,482.65 (430.77 USD) returns from fish sales. Furthermore, significant mean differences in membership to cooperatives are reported, with 65% of CIG having membership and just 47% of Non-CIG households having membership.

**Table 2:** Differences in characteristics of aquaculture farmers

	CIG members		Non − CIG members		
	Mean	Std. err	Mean	Std. err	Diff
Returns from fish sells (Ksh)	81,514.77	5564.4	55,482.6	5395.4	− 26,032^**^
Fish yields at harvest (kg)	3268.05	1019.20	2883.479	96.39	− 384.57
Education	13.519	0.1278	13.877	0.2014	0.3668
Gender	0.8874	0.0141	0.8950	0.0208	0.0076
Dist to main fish market	0.8937	0.0998	3.7188	2.2872	2.8251
Membership to cooperatives	0.6581	0.0211	0.4703	0.0338	− 0.187^***^
Credit	0.4644	0.0222	0.4795	0.0338	0.0150
Fingerlings stocked	3162.4	359.2	2274.5	140.9	− 887.9
Number of ponds	2.296	0.170	2.57	0.162	0.273
Hormonal sex reversal	0.5584	0.0221	0.6073	0.0331	0.048
Selective breeding	0.6877	0.0206	0.6697	0.0319	− 0.018
Feed regime	0.9526	0.0095	0.9361	0.0165	− 0.016
Extension access	0.8310	0.0167	0.5205	0.0338	− 0.310^***^
Support from crop farming projects	0.3953	0.0218	0.3789	0.0329	− 0.016
What are the number of times trainings/advice received	3.130	0.1528	3.4312	0.5026	0.301
Cage size (m^3^)	581.2	128.4	239.0	16.25	− 342.1

*p < 0.05; **p < 0.01; ***p < 0.001

In Table 2, we report significant differences in access to extension services. The findings revealed that 83.10% of CIG members accessed extension services, compared to only 52% of CIGs. Information provided during extension visits and farmers field school act proactive steps of building farmers’ capacity, and dissemination of agricultural technologies (Gichuki et al. 2023). Furthermore, households with CIG membership had larger cages and stocked more fingerlings than their counterparts who did not have membership to CIGs. Cage culture has emerged as a crucial method for individuals with minimal means and expertise to engage in aquaculture and produce substantial amounts of fish for personal and non-personal consumption. Cages can be built using readily accessible materials with low financial commitment and positioned in small ponds or public bodies of water (Fitzsimmons et al. 2011). The descriptive comparisons seem to suggest that ABDP interventions to common interest groups would play a significant role in fish yields. However, the findings in Table 2 cannot be used to make inferences as the simple comparison of mean differences does not account for confounding factors such as observed household and farm-level characteristics.

### Empirical results and discussion

The estimates of the factors that influence a farmer’s decision to join CIGs and the impact of participating ABDP on returns from tilapia fish sells and fish yields are presented in Tables 3 and 4. As indicated previously, the FIML approach estimates both the selection and outcome equations jointly. The selection equations that represent the determinants of CIG membership are given in the second column of Tables 3 and 4. The outcome equations that represent the impact of ABDP on returns from fish sells and fish yields harvested, for both CIGs members and non-members, are given in the third and fourth columns of Tables 3 and 4. In addition, the estimates of the residuals derived from the first-stage regression for the potential endogenous variables that include trust in farmers groups (Res) and extension access (Res) are also presented in the second columns of Tables 3 and 4.

**Table 3:** CIG membership and ABDP impact on tilapia fish returns

			Returns from fish sells			
	Selection		CIG members		Non-CIG members	
	Coefficient	Std. err	Coefficient	Std. err	Coefficient	Std. err
_cons	0.401	0.425	607.7	441.8	896.8	5869
Age	− 0.007	0.004	− 884.2^**^	368.0	− 103.9^*^	476.6
Household size	0.011^**^	0.017	200.4	177.6	201.4	205.1
Education	0.031^*^	0.018	103.3^**^	175.1	− 311.3	283.6
Gender of HH	0.138	0.149	175.7^*^	15,767.1	265.4	199.6
Distance to main fish market	− 0.031^*^	0.019	− 187.1	203.8	8.798	175.1
Credit	0.078	0.096	232.9^**^	100.1	− 16,841	12,837
Number of ponds	− 0.010^***^	0.014	121.7^***^	151.6	245.4	287.4
Cage size (m^3^)	0.001^***^	0.000	8.655^***^	1.985	0.642	33.06
Membership to cooperatives	0.088^***^	0.099	310.2^**^	104.9	392.9^**^	133.8
Selective breeding	− 0.052^*^	0.124	− 148.5	130.6	4654	15,042
Hormonal sex reversal	0.298	0.202	155.5	220.7	− 135.1	257.6
Support from crop farming projects	− 0.040	0.097	− 225.2	101.1	203.5	135.4
Feed regime	− 0.030	0.114	661.4^***^	119.7	492.7	154.9
Fingerlings stocked	0.007^***^	0.001	7.629	6.270	− 4.945	3.421
Production cycle of Tilapia in months	− 0.055	0.026	− 426.3	278.2	− 1639	3326
Trust in farmers groups (Res)	− 0.084^***^	0.279				
Extension access (Res)	0.321^***^	0.066				
Ln *σ*1, Ln*σ*2			11.69^***^	0.039	11.30^***^	0.056
*ρ*1, *ρ*2			− 0.542^***^	0.104	0.146	0.216
LR test of indep. eqns	191.93^***^					
Log likelihood	− 8503.2					
Observation	724					

*p < 0.05; **p < 0.001; ***p < 0.001

**Table 4:** CIG membership and ABDP impact on tilapia fish yields

			Fish yields at harvest			
	Selection		CIG members		Non-CIG members	
	Coefficient	Std. err	Coefficient	Std. err	Coefficient	Std. err
_cons	0.313	0.448	113.9	134.1	1826.1	1018.7
Age	0.001^*^	0.004	2.899^*^	11.15	− 1.005	8.616
Household size	− 0.010^***^	0.018	30.65^**^	54.21	4.773	38.17
Education	0.023	0.018	45.68	52.84	4.587	51.45
Gender	− 0.001	0.155	− 3.064	477.6	18.34	367.8
Dist to main fish market in KM	− 0.012^*^	0.018	− 67.28^*^	59.94	− 2.961	3.303
Credit	0.040	0.099	− 25.25	303.6	− 52.57	236.6
Number of ponds	0.030^***^	0.015	− 64.3	45.71	− 18.85	52.28
Cage size (m^3^)	0.001^***^	0.007	0.058^**^	0.060	0.430	0.568
Membership to cooperatives	0.142^***^	0.103	386.3^**^	318.7	120.2	246.2
Selective breeding	0.025^***^	0.131	173.4^*^	397.5	278.9^*^	279.1
Feed regime	0.201	0.212	58.74	67.2	− 270.9^*^	476.5
Hormonal sex reversal	− 0.040	0.102	− 269.9	309.6	− 32.26	249.6
Support from crop farming projects	0.020	0.116	83.44	363.2	− 162.6	282.2
Number of fingerlings stocked	0.028^***^	0.001	− 0.032	0.022	0.148	0.060
Production cycle of Tilapia in months	− 0.010	0.027	20.16	85.07	− 23.93	59.23
Trust in farmers groups (Res)	− 0.100^***^	0.035				
Extension access (Res)	0.451^***^	0.086				
Ln *σ*1, Lnσ2			8.153^***^	0.034	7.203^***^	0.178
*ρ*1, *ρ*2			4.155^***^	0.395	0.097	2.605
LR test of indep. eqns	312.85^***^	120.3				
Log likelihood	− 6029.4	239.61				
Observation	724					

*p < 0.05; **p < 0.01; ***p < 0.001

### Determinants of benefiting ABDP on *tilapia* returns

The selection specification presented in Table 3 shows that household size and education years of the household determined membership to CIG. The cage size (m^3^) and number of pond variable are positive and significantly different from zero, suggesting that farmers with larger cage size and more ponds were more likely to have CIG membership benefit from ABDP program interventions. Development programs primarily focus on increasing wages as a strategy to reduce poverty, while also emphasizing the importance of production assets in the broader context of poverty alleviation (Johnson, et al. 2016). The study found that the variable for membership in cooperative is positive and significant. Smallholders can improve their competitiveness in dynamic market by utilizing cooperatives (Fischer and Qaim 2014). Furthermore, the establishment of co-operative is regarded as a potential institutional remedy to address the challenges of elevated transaction costs and market failure in developing nations.

The coefficients of the variable representing household characteristics, specifically years of education and male-headed households, were found to positively and significantly influence the fish net return for CIG members (ABDP beneficiaries). These findings are consistent with previous research highlighting significant gender disparity in farm management and asset ownership, reflecting existing gender norms that limit women’s ability to invest in more profitable subsistence methods like market-oriented agriculture (Deere and Doss 2006; Doss et al. 2020). Additional research indicates that there is a lack of emphasis on the correlation between the asset endowment of women and their capacity to engage in and derive advantages from agricultural interventions (Quisumbing et al. 2015). The variable representing membership to cooperatives shows a significant and positive impact on fish returns for both CIGs members and nonmembers, suggesting that cooperatives are a vital determinant of higher incomes from fish. These findings are consistent with Fischer and Qaim (2014) which suggests that cooperative membership can help farmers access sufficient market information, consolidate their farm production, and identify the right markets that offer more favorable pricing. Similarly, Abdulai and Huffman (2014) observed that cooperative membership influences market access, technology adoption, pricing, and income levels.

The number of ponds and cage size owned have positive and significant impact on the fish net returns for CIGs members. Khan et al. (2021) observed similar findings: the profitability of fish per hectare grows as the size of the farm increases, although not at the same rate as the increase in farm size. The variable representing credit access has positive and significant effect on the incomes from fish returns for CIGs members.

Intensive or semi-intensive commercial aquaculture necessitates a sufficient provision of adequate supply of feeds, fingerlings, labor, machinery, and medicines, particularly during culture periods. Obtaining these inputs necessitates a substantial amount of financial resources, and small-scale farmers encounter institutional limitations, specifically, restricted availability of financing (Mitra, et al. 2023). The majority of aquaculture farms are of modest size and lack the financial resources to get these expensive ingredients (Odende, et al. 2022). Development initiatives incorporate interventions that establish connections between farmers and microcredits in order to provide financial support for farming activities (Jindo et al. 2023; Muringai et al. 2021).

Another important finding in Table 3 is the variance parameters; the significant and high values of Ln *σ*1 and Ln *σ*2 indicate a notable variance in the outcomes, suggesting considerable differences in the factors affecting the net returns and productivity between CGI members and non- CIGs members. Further observation reveals that the coefficient *ρ*1 is negative and statistically significant, suggesting that there is a negative correlation between unobserved factors influencing participation in CIGs and the outcomes (net returns). This implies that farmers who choose to participate in CIGs might have unobserved characteristics that negatively impact tilapia fish net returns, but CIG membership helps offset this. The coefficient *ρ*2 is not statistically significant, indicating no significant correlation between unobserved factors and outcomes for non- CIG participants. This suggests that unobserved characteristics of non-participating farmers do not significantly influence their net returns and productivity. The significant LR test statistic indicates that the participation decision and outcome equations are not independent, justifying the use of the endogenous switching regression model. This result confirms that selection bias exists, and farmers’ decisions to participate in CIGs are correlated with unobserved factors that also affect tilapia returns.

### Determinants of benefiting ABDP *tilapia* fish yields produced

Table 4 presents the estimates of the impact of CIGs membership and the impact of ABDP program on the fish yield production. The selection specifications presented in collum 2 in Table 4 show that age of the household head and the size of the household were significant determinants on joining CIG. The estimate variables: number of ponds and the size of cages, were positive and significant implying that the variables determined farmers’ participation in CIGs. These findings suggest that assets owned by farmers have the ability to shape the design, execution, and results of programs by determining the individuals who engage (or do not engage) in them, as well as the manner and extent to which they derive benefits (Mark et al. 2020). Selective breeding of fish was positive and significant for farmers decision to participate in CIGs. Agricultural interventions can also introduce improved technologies or institutional innovations that increase the returns to the productive assets used in agriculture-based livelihood strategies, potentially raising the returns (Johnson et al. 2016). The study findings also show that aquaculture trainings and advice on agri-business determined farmers participation in CIGs. Likewise, the coefficients of the variables representing participation in cooperatives are positive and significant and thus seem to be an important determinant of participating in CIGs. The establishment of farmers’ cooperative organizations is advocated in aquaculture development as viable strategies to foster the participation of small and medium-scale commercial farmers (Stutzman et al. 2017).

Table 4 illustrates the effects of CIG membership and the benefits of the ABDP on fish yields. The findings reveal that the use of selective breeding technology had a significant and positive impact on the fish yields of CIG members, whereas this effect was not observed among Non-CIG members. In their study, Saha et al. (2022) examined the implementation of scientific aquaculture management and found that adopting such approaches leads to higher levels of productivity and profitability. Obiero et al. (2019) observed similar findings, discovering that the likelihood of adopting aquaculture technologies is positively associated with projected better yields.

Additional findings indicate that involvement in cooperatives has a favorable and statistically significant effect on the quantity of fish harvested by CIG members. Cage size had a positive and significant impact on the fish yields for the CIG members had benefited from ABDP program interventions. Khan et al.’s (2021) study revealed comparable observations that as farm size increases, so does fish productivity, as measured by quantity produced per hectare.

The estimated variance parameters Ln *σ*1 and Ln *σ*2 are presented in the lower portions of Table 4. The significant variance parameters indicate that there is considerable heterogeneity in fish yields within each group, meaning that unobserved factors (like farm management practices, etc.) significantly impact the outcomes for both CIG and Non-CIG members. In addition, the findings reveled that *ρ*1 positive and highly significant correlation coefficient suggests a strong positive correlation between unobserved factors that influence both the decision to join CIGs and the outcomes (fish yields). Essentially, unobserved characteristics that lead farmers to join CIGs also positively influence their fish yields. LR test statistic is highly significant, which rejects the null hypothesis that the equations for participation and outcomes are independent. This result confirms that there is selection bias in the model, meaning the decision to join CIGs is not random but is instead correlated with the factors that also affect fish yields. The econometric observations can be interpreted to imply the presence of selection bias (Ma and Abdulai 2016). Thus, failing to correct for selectivity effects may give biased coefficients of the results. As a result, accounting for both observable and unobservable elements is critical to achieving unbiased treatment effects (ATT).

The estimates for the average treatment effects on the treated (ATT), which shows the causal effects of CIG membership and benefits of ABDP program interventions on fish yields and fish returns, are presented in Table 5. The results are derived after ESR model, as illustrated in Eq. (8) and account for selection bias arising from both observable and unobservable factors. The ATT indicates that CIG members earned, on average, an additional income of 26,647.73 Ksh (205usd) compared to what they would have earned had they not been members. This suggests that CIG membership has a significant positive effect on fish returns income. The ATT represents a 32.3% increase in income for CIG members, demonstrating that CIG membership substantially boosts income from fish farming. The positive *t*-value and significance level indicate that this impact is statistically significant. The ATU shows that Non-CIG members would have seen their income increase by 2017.345Ksh (USD 15.5) if they had joined CIGs. The ATT for fish yields indicates that CIG members produce an average of 17.590 kg more fish than they would have if they were not members.

**Table 5:** Impact of ABDP on tilapia fish income and fish harvested ATT and ATU

Outcome variable	Mean outcome				ATT	ATU	*t*-value	% change
	**CIG members**		**Non-CIG members**					
Fish returns income	81,514.77	125,168.7	55,482.65	79,662.39	26,647.73	2017.345	2.14^***^	32.3%
Fish yields	3362.658	7291.982	2883.479	1426.57	17.590	14.245	1.38^***^	6.6%

* p<05; ** p<.01; *** p<.001

The results reveal that the participation CIG and benefiting from ABDP interventions tends to significantly increase income from fish. Ahmed et al. (2010) demonstrates that while fish production can be profitable, greater net profits are achieved through intensive mode production as opposed to semi-intensive or small-scale mode production systems. The causal effect of CIG membership and benefits of ABDP on fish yields is much higher than from Non-CIG members, with a 6.6% percent increase in fish yields. The findings indicate that implementing interventions such as fish cages, fingerlings, advice in aquaculture production and marketing can enhance farmers' well-being by increasing their revenue.

## Conclusions and policy implications

This study provides critical insights into the impact of participation in Common Interest Groups (CIGs) and aquaculture development support programs on the productivity and net returns of tilapia fish farmers. Using data collected from 506 CIG members and 218 Non-CIG members in Western Kenya, the analysis revealed significant differences in earnings, cooperative membership, and access to extension services between CIG farmers and Non-CIG members. These descriptive differences underscored the need for a robust analytical approach to address potential selection bias.

To accurately assess the impact of CIG participation, an endogenous switching regression (ESR) model was employed, allowing the study to account for both observed and unobserved factors influencing farmers' decisions to join CIGs. The results demonstrated that CIG membership and support from the Aquaculture Business Development Program (ABDP) interventions significantly improved fish yields and income returns. Specifically, CIG participation resulted in a 32.3% increase in fish returns and a 6.6% increase in fish yields. These findings emphasize the effectiveness of CIGs and targeted development interventions in enhancing the livelihoods of small-scale aquaculture producers.

The analysis highlighted that several factors, including the quantity of fingerlings stocked, cage size, cooperative membership, selective breeding techniques, number of ponds, and access to aquaculture and agribusiness training, significantly influenced farmers' decisions to participate in CIGs. The ESR model's results further suggested that CIG members are likely to have inherent advantages, such as better access to resources and networks, that contribute to their improved outcomes.

The findings of this study carry several important policy implications that can inform strategies to enhance the productivity and profitability of small-scale aquaculture. The study underscores the significant benefits of CIG membership, suggesting that policies should focus on encouraging and facilitating farmer participation in these groups. CIGs provide a platform for knowledge sharing, collective action, and access to resources that individual farmers may lack. Policies that support the formation and sustainability of CIGs can help amplify these benefits, thereby boosting aquaculture production and income. Membership in cooperatives was shown to positively impact fish yields and income returns. Cooperatives can serve as critical platforms for skill enhancement, knowledge exchange, and innovation. Policymakers should support the development of cooperatives, facilitate their access to markets, and promote linkages with agribusiness corporations. By doing so, cooperatives can secure better prices, reduce transaction costs, and improve the overall bargaining power of farmers.

The study highlights the role of training and technical advice in enhancing aquaculture outcomes. Policymakers should invest in capacity-building programs that focus on aquaculture best practices, selective breeding, pond management, and business skills. Establishing continuous learning platforms for farmers, such as workshops, field demonstrations, and extension services, can sustain the benefits of development projects like ABDP even after the projects conclude. The significant positive effects of fingerling stocking density, cage size, and selective breeding on productivity suggest that access to quality inputs and technologies is crucial for improving yields. Policies should be aimed at reducing barriers to accessing these inputs through subsidies, credit facilities, or partnerships with private sector suppliers. Facilitating access to innovative technologies and best practices will enable farmers to optimize production and improve returns.

The study finds that benefits from development projects such as ABDP are often lost once project support ends. To ensure the sustainability of these benefits, policymakers should focus on integrating farmers into broader value chains, strengthening their connections with local supply chain actors, and coordinating services among different stakeholders. Establishing networks of fee-based field agents who provide ongoing support can also help maintain gains beyond project timelines. Public–private partnerships can play a pivotal role in enhancing aquaculture development by bringing together the expertise, resources, and networks of various actors. Encouraging such collaborations can improve access to markets, technology, and finance, creating a more supportive environment for small-scale aquaculture farmers.

In conclusion this study highlights the transformative potential of CIGs and targeted aquaculture support programs in improving the productivity and incomes of small-scale fish farmers. By addressing key barriers such as access to inputs, training, and market linkages and by promoting cooperative and CIG participation, policymakers can enhance the resilience and profitability of aquaculture enterprises. Sustained support and strategic investments in aquaculture infrastructure and capacity building will be crucial in leveraging these gains to reduce rural poverty and drive economic growth in Kenya’s aquaculture sector.

## Data Availability

Sequence data that support the findings of this study have been deposited in the .
